# Supplementary material for: Semen inhibits Zika virus infection of cells and tissues from the anogenital region
Source: Nat Commun. 2018 Jun 7;9:2207. doi: 10.1038/s41467-018-04442-y (PMC5992203; doi:10.1038/s41467-018-04442-y)
Supplement: Supplementary file 1 — Supplementary Information [file 41467_2018_4442_MOESM1_ESM.pdf]

Müller *et al.*

Semen Inhibits Zika Virus Infection of Cells and Tissues from the Anogenital Region

Supplementary Information

Supplementary Figures

Supplementary Figure 1

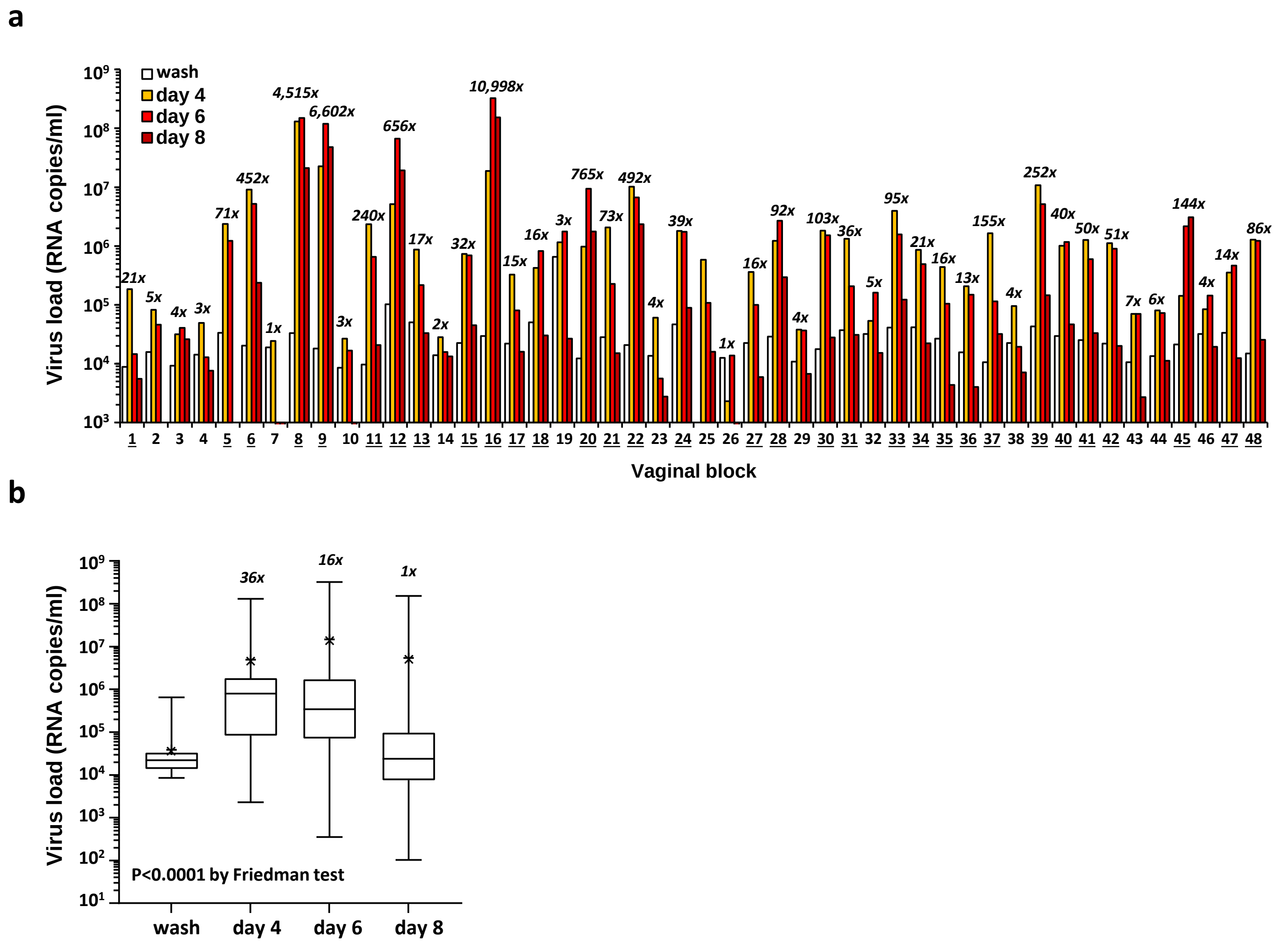

Supplementary Figure 1. ZIKV infects and replicates in vaginal tissue. a) 48 vaginal tissue blocks from one donor were incubated with ZIKV GWUH for 2 hours, washed with PBS, and then each transferred into individual wells of a 96-well plate. Aliquots of the supernatants were collected at days 0 (wash control), 4, 6, and 8, and viral genome copy numbers were determined by qPCR. Maximum fold-increase in titers were compared to the wash control, and shown in italics above the corresponding bars. Underlined are vaginal blocks where titers increased ≥ 10 fold. b) Box plot representation of the median (± maximum and minimum titer) of the 48 measured blocks. Black star indicates averages.

Supplementary Figure 2

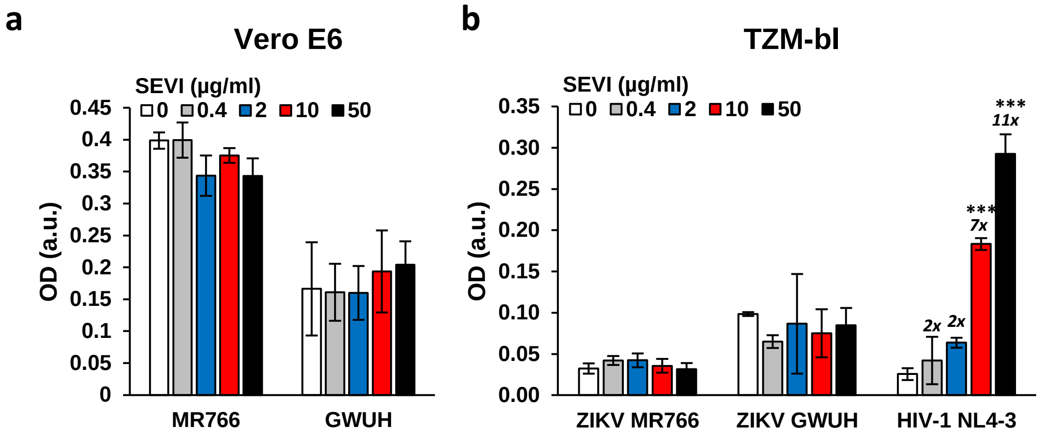

Supplementary Figure 2. SEVI enhances HIV-1 but not ZIKV infection. a) ZIKV MR766 or GWUH were incubated with increasing concentrations of SEVI and then added to Vero E6 cells. Two days later infection rates were determined by a cell-based ZIKV immunodetection assay that enzymatically quantifies the flavivirus protein E. b) ZIKV MR766, GWUH, or HIV-1 NL4-3 92TH014.12 were incubated with SEVI and then added to TZM-bl cells and infection determined two days later by a cell-based ZIKV protein E or HIV-1 p24 antigen immunodetection. Data represent average values obtained from triplicate infections ± standard deviations. Fold higher infection rates compared to the absence of SEVI are indicated above the corresponding bars in italics. OD optical density, a.u.: arbitrary units, * P<0.01, ** P<0.001, *** P<0.0001 (by one-way ANOVA with a Bonferroni post-test).

Supplementary Figure 3

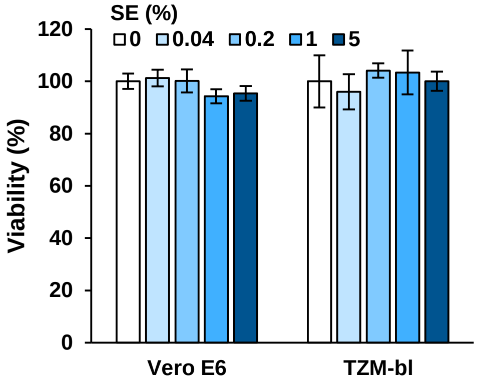

Supplementary Figure 3. Cytotoxicity of semen to Vero E6 or TZM-bl cells. Vero E6 or TZM-bl cells were incubated with indicated SE concentrations for 2 hours at 37°C after which medium was changed. Two days post treatment, the CellTiter-Glo® luminescent cell viability assay was performed. Viability was normalized relative to enzyme activity in absence of SE. All data represent average values obtained from triplicates ± standard deviations. No significant difference between samples was detected using a one-way ANOVA with a Bonferroni post-test.

Supplementary Figure 4

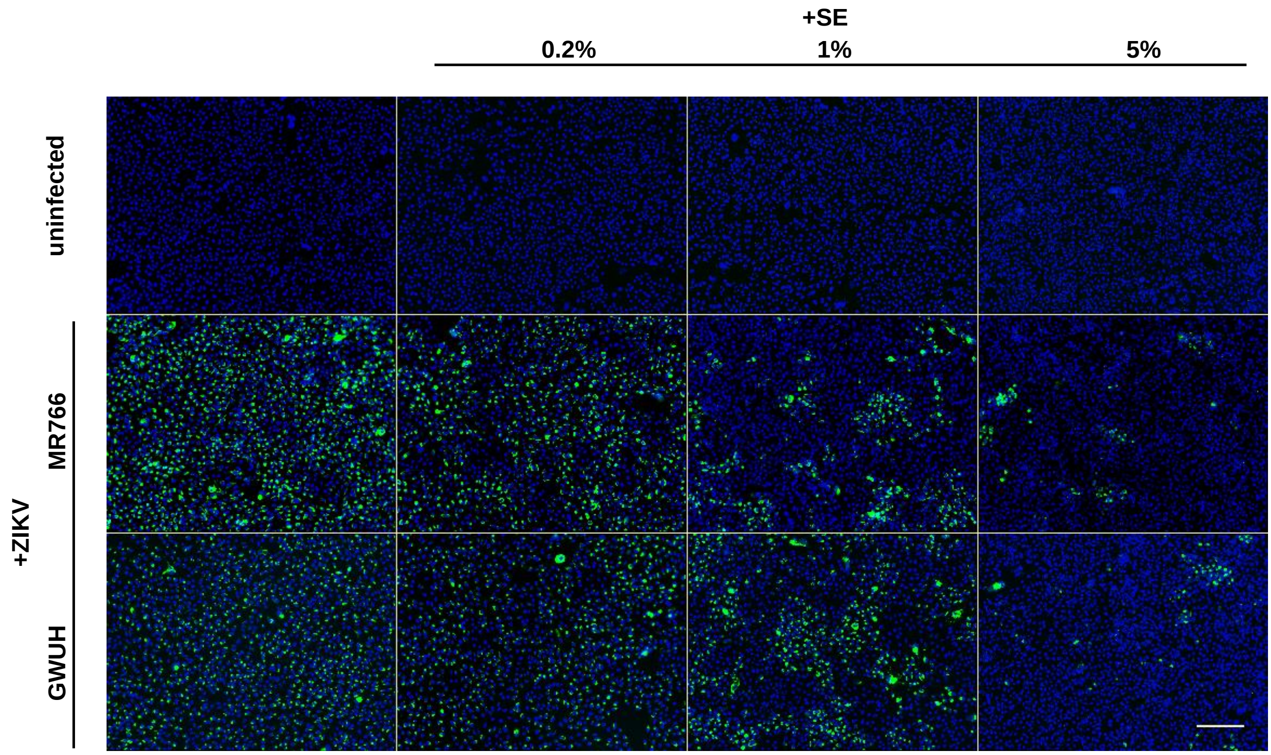

Supplementary Figure 4. Semen reduces the number of ZIKV infected cells as detected by fluorescence microscopy. Vero E6 cells were incubated with indicated concentrations of semen (SE) before inoculation with ZIKV MR766 or GWUH. At 2 days post-infection cells were stained for protein E (green) and nuclear DNA (blue) and cells were visualized by fluorescence microscopy. Scale bar corresponds to 200 µm. For quantification of microscopy data, see Fig. 3d.

Supplementary Figure 5

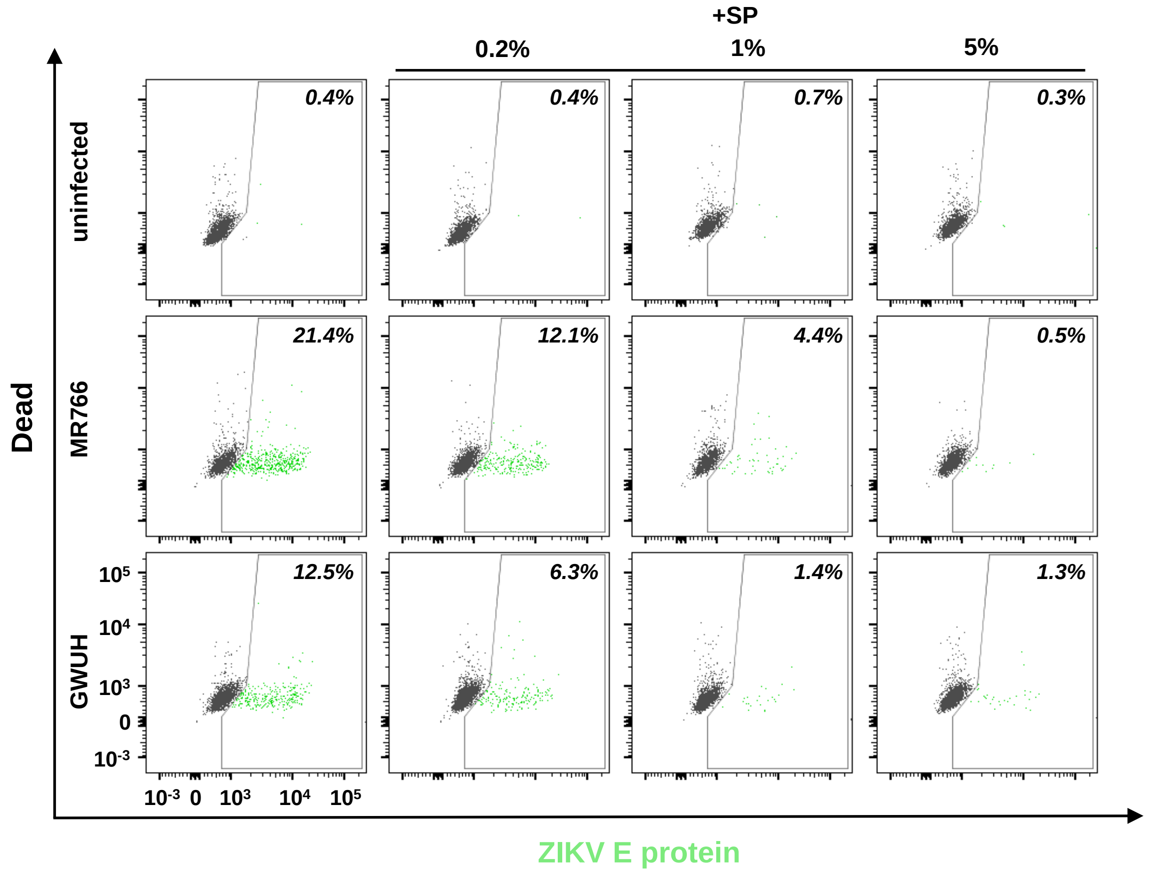

Supplementary Figure 5. Seminal plasma reduces the number of ZIKV infected cells as detected by flow cytometry. Vero E6 cells were incubated with indicated concentrations of SP before inoculation with ZIKV MR766 or GWUH. Two days post inoculation cells were stained with a viability marker and for ZIKV protein E, and then analyzed by flow cytometry. Percentage of infected cells is indicated in italics in the corresponding boxes. For quantification of mean fluorescence intensity, see Fig. 3e.

Supplementary Figure 6

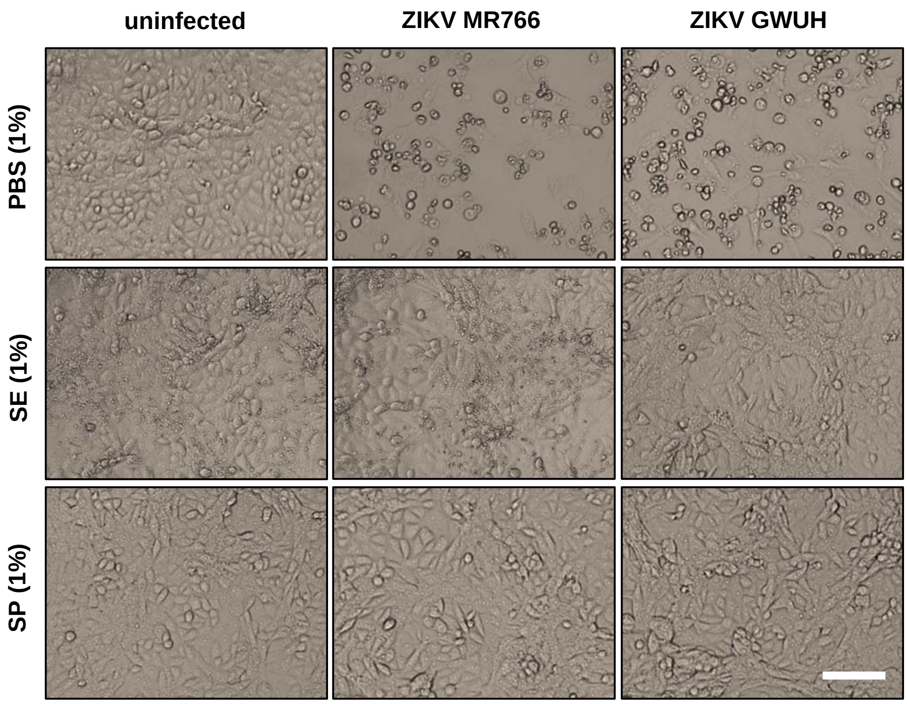

Supplementary Figure 6. Semen and seminal plasma prevent the ZIKV-induced cytopathic effect. Vero E6 cells were incubated with 1% PBS, SE or SP before inoculation with ZIKV MR766 or GWUH. Images were taken 5 days post-infection. Scale bar corresponds to 100 µm.

Supplementary Figure 7

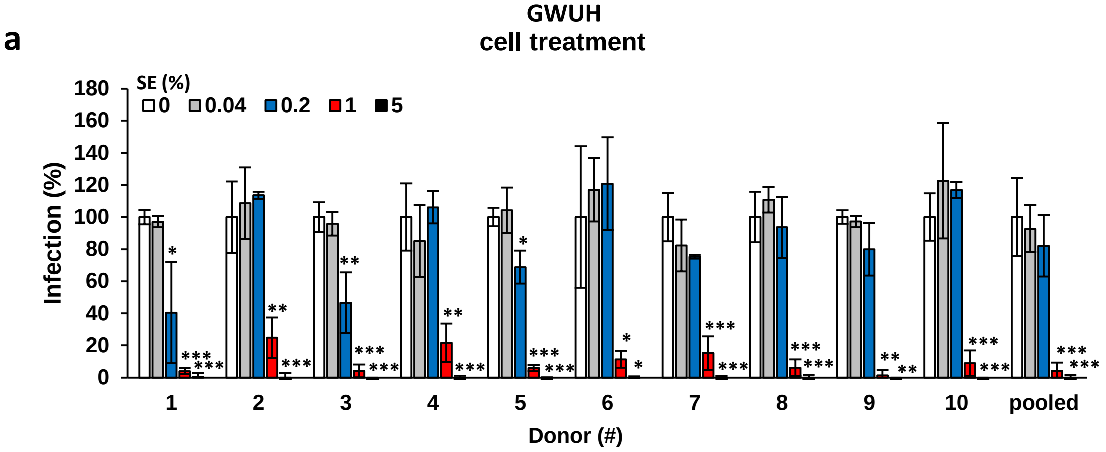

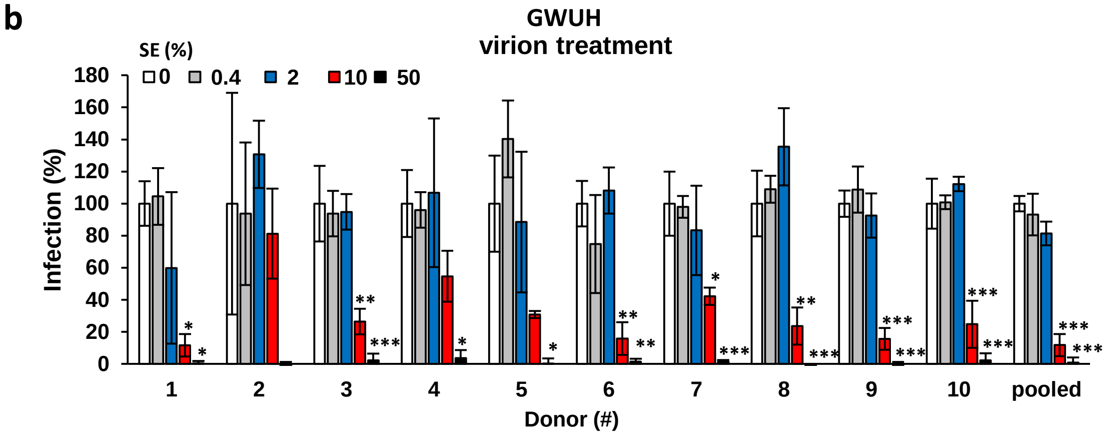

Supplementary Figure 7. Semen-mediated ZIKV inhibition is not donor-specific. a) Vero E6 cells were incubated for 10 min with the indicated concentrations of SE derived from 10 individual donors, or a pool of all 10 samples ("cell treatment"). Cells were then inoculated with ZIKV MR766 and monitored for infection levels. b) ZIKV MR766 was first incubated with 0, 0.4, 2, 10 or 50% of the individual and pooled SE samples for 10 minutes ("virion treatment") and then added to Vero E6 cells resulting in similar final SE concentrations as shown in (a). Infection rates were determined by a cell-based ZIKV immunodetection assay 2 days post-infection. Data are normalized to infection in absence of SE and represent average values obtained from triplicate infections ± standard deviations. For IC_{50} values, see Table 2. * P<0.01, ** P<0.001, *** P<0.0001 (by one-way ANOVA with a Bonferroni post-test).

Supplementary Figure 8

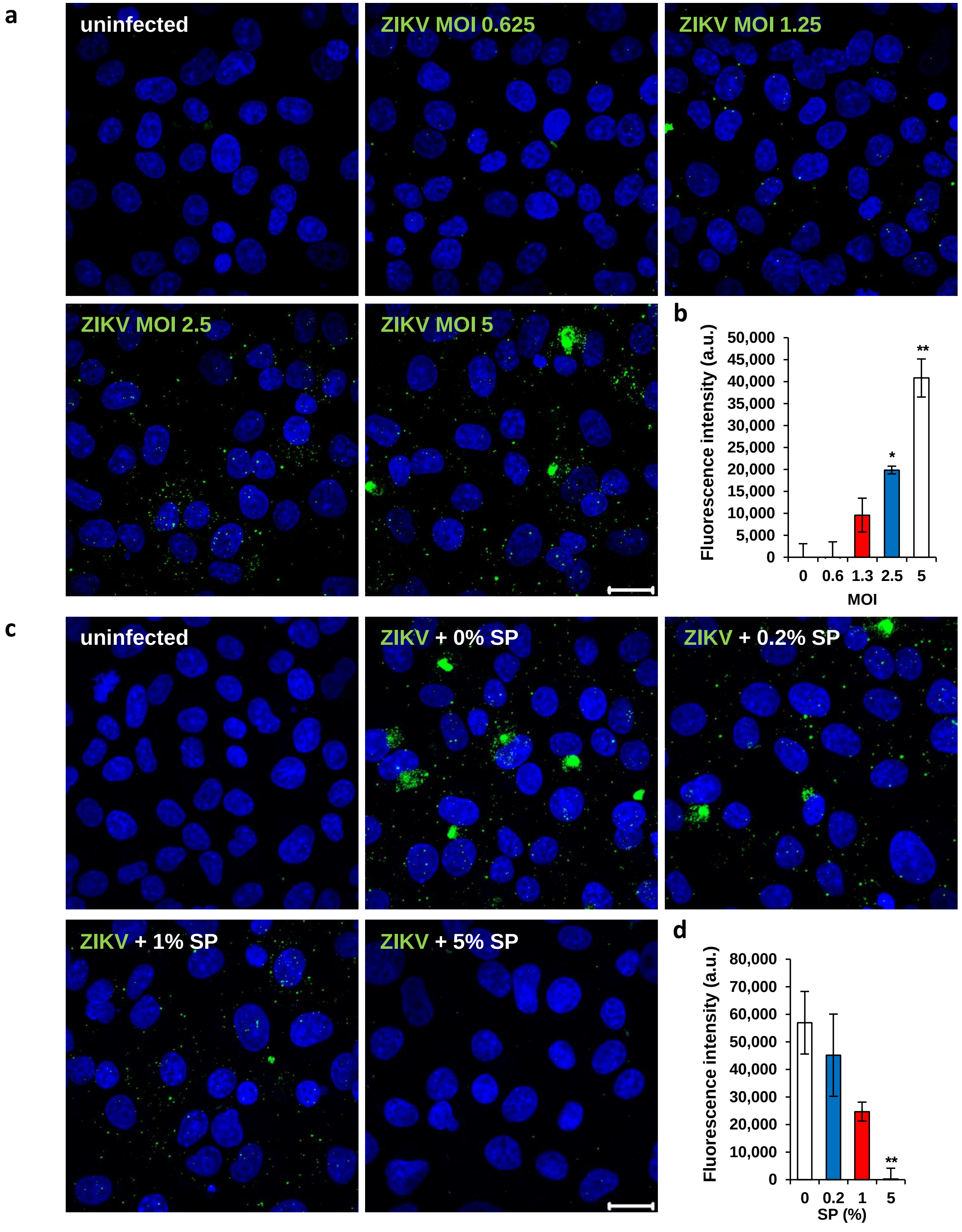

Supplementary Figure 8. SP inhibits attachment of Zika virions to cells at 37°C. a) Vero E6 cells were incubated with increasing MOIs of ZIKV MR766 for 2 hours at 37°C. Cells were then washed, fixed, permeabilzed and stained for flavivirus protein E (green) and cell nuclei (blue). A z-stack of 14 confocal microscopy images was taken and combined to a maximum intensity projection. Contrast of the green signal was increased to facilitate visualization of viral particles. Scale bar is 20 µm. b) Signal intensity of the projections in (a) was quantified from three z-stacks ± standard deviation. c) Zika virions were allowed to attach to Vero E6 cells in presence of the indicated concentrations of SP for two hours at 37°C. A z-stack of 14 confocal microscopy images was taken and combined to a maximum intensity projection. Contrast of the green signal was increased to facilitate visualization of viral particles. Scale bar is 20 µm. d) Protein E fluorescence of (c) was quantified from three z-stacks ± standard deviation (see Fig. 4d). a.u.: arbitrary units, * P<0.01, ** P<0.001 (by one-way ANOVA with a Bonferroni post-test).

Supplementary Figure 9

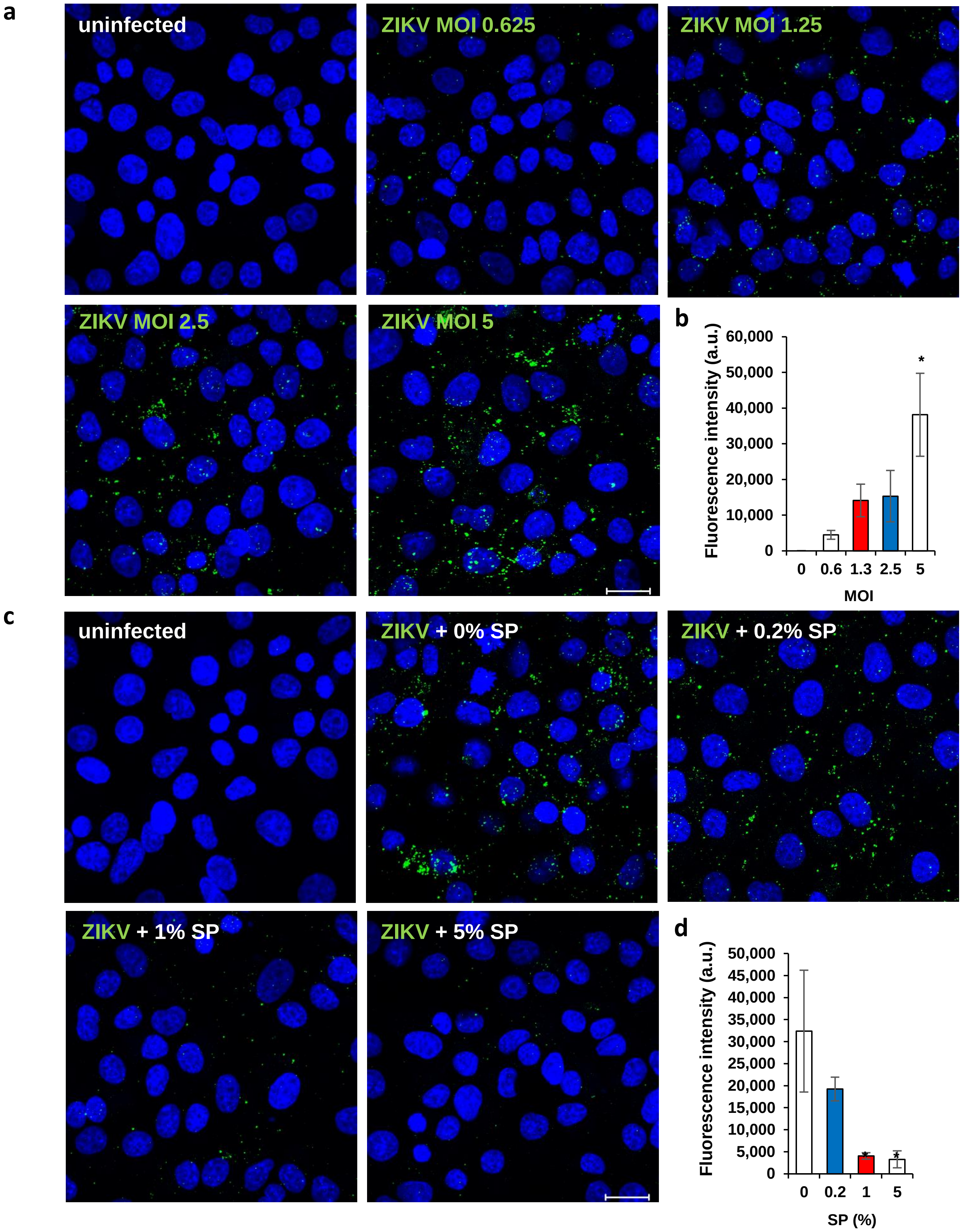

Supplementary Figure 9. SP inhibits attachment of Zika virions to cells at 4°C. a) Vero E6 cells were incubated with increasing MOIs of ZIKV MR766 for 2 hours at 4°C. Cells were then washed, fixed, permeabilzed and stained for flavivirus protein E (green) and cell nuclei (blue). A z-stack of 14 confocal microscopy images was taken and combined to a maximum intensity projection. Contrast of the green signal was increased to facilitate visualization of viral particles. Scale bar is 20 µm. b) Signal intensity of the projections in (a) was quantified from three z-stacks ± standard deviation. c) Zika virions were allowed to attach to Vero E6 cells in presence of the indicated concentrations of SP for two hours at 4°C. A z-stack of 14 confocal microscopy images was taken and combined to a maximum intensity projection. Contrast of the green signal was increased to facilitate visualization of viral particles. Scale bar is 20 µm. d) Protein E fluorescence of (c) was quantified from three z-stacks ± standard deviation (see Fig. 4e). a.u.: arbitrary units, * $P<0.01$, (by one-way ANOVA with a Bonferroni post-test).

Supplementary Figure 10

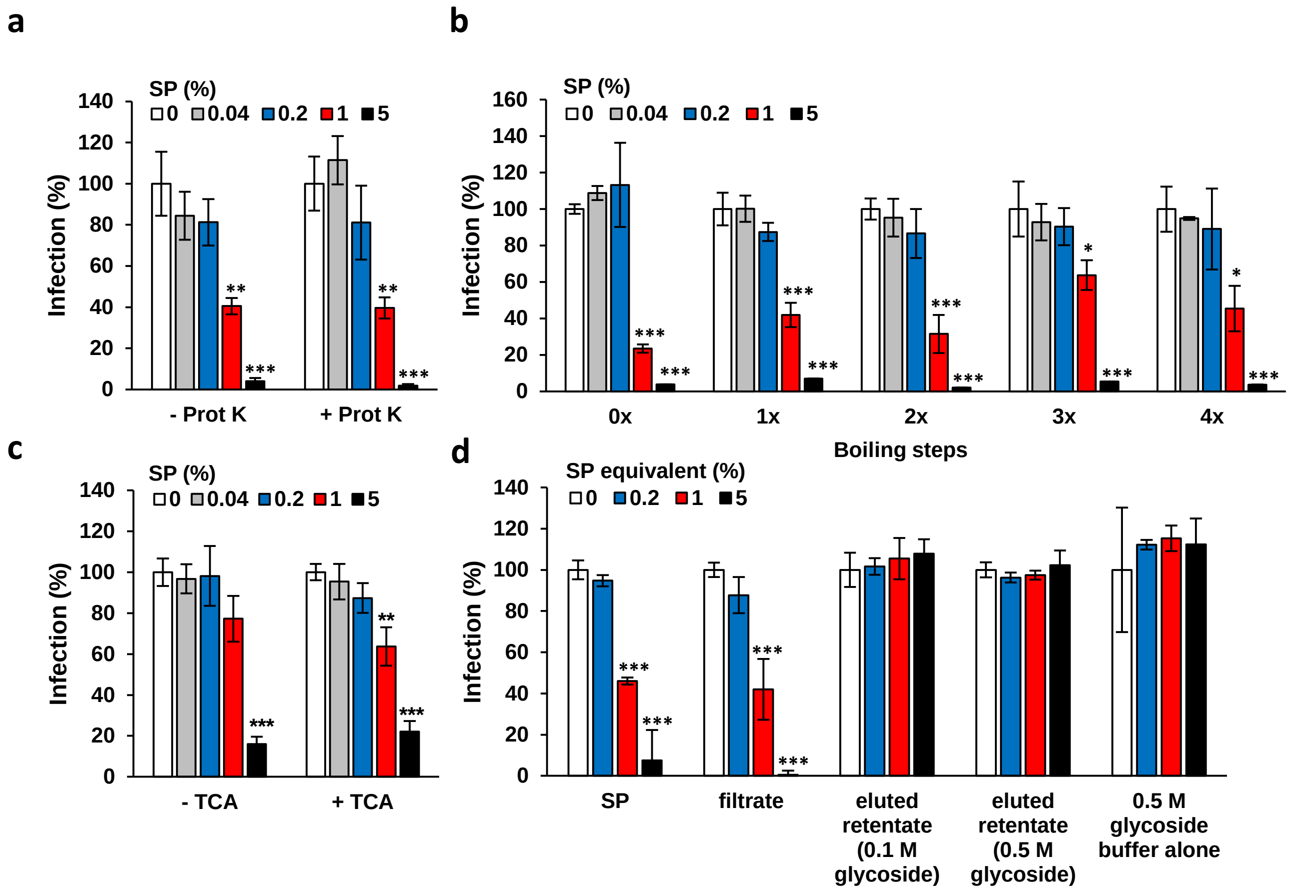

Supplementary Figure 10. ZIKV-inhibition by semen is not mediated by protein or glycosylated structures. a) 50% SP was incubated in absence (-) or presence (+) of proteinase K for 5 hours at 37°C and then proteinase K activity stopped by 20 min denaturation at 99°C. Denatured protein was centrifuged and discarded. Treated samples were used to incubate Vero E6 cells at indicated concentrations for 10 min before inoculation with ZIKV MR766. After 2 days, infection was determined by cell-based ZIKV immunodetection assay that enzymatically quantifies the flavivirus protein E. Average infection rates are normalized to the corresponding infection averages in the absence of SP. Data represent average values obtained from triplicate infections ± standard deviations. b) 50% SP was incubated for 20 min at room temperature (0x) or boiled one to four (1-4x) times at 99°C. In between incubations, denatured protein was centrifuged and discarded. ZIKV infection in the presence of treated samples was monitored as described in (a). c) SP proteins were precipitated by 1 h incubation with 10% TCA (+) or 10% PBS (-) as a control. Supernatants were then pH adjusted with NaOH. ZIKV infection in the presence of treated samples was monitored as described in (a). d) 50% SP was applied to a Con A Sepharose 4B column. The retentate was eluted by glycoside buffers, buffer exchanged and diluted to the volume of the filtrate with PBS. ZIKV infection in the presence of treated samples was monitored as described in (a). * $P<0.01$, ** $P<0.001$, *** $P<0.0001$ (by one-way ANOVA with a Bonferroni post-test).

Supplementary Figure 11

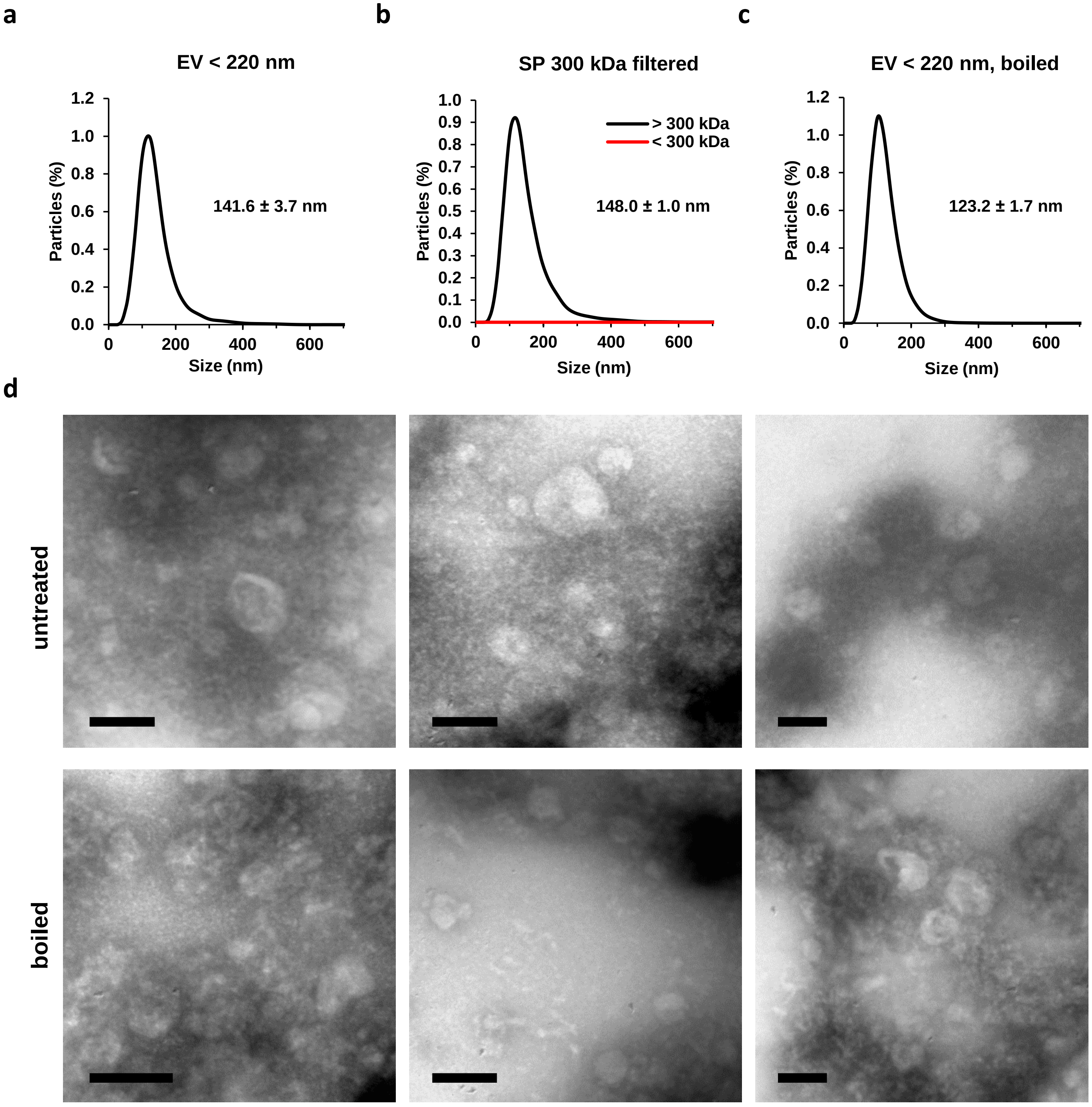

Supplementary Figure 11. Semen EVs are in average 142 nm in diameter. a) SP derived EVs (< 220 nm) (see Fig. 5c) or b) the filtrate and retentate of 0.2 µm filtered SP applied to a 300 kDa molecular weight filter (see Fig. 5b) or c) the 99°C boiled EVs (see Fig. 5d) were analyzed for their particle size distribution via nanoparticle tracking analysis using a NanoSight LM10. Data are averages of three tracking measurements for the duration of 60 s. d) Boiled or untreated 0.2 µm filtered SP was negatively stained and imaged by transmission electron microscopy. Scale bars are 100 nm.

Supplementary Figure 12

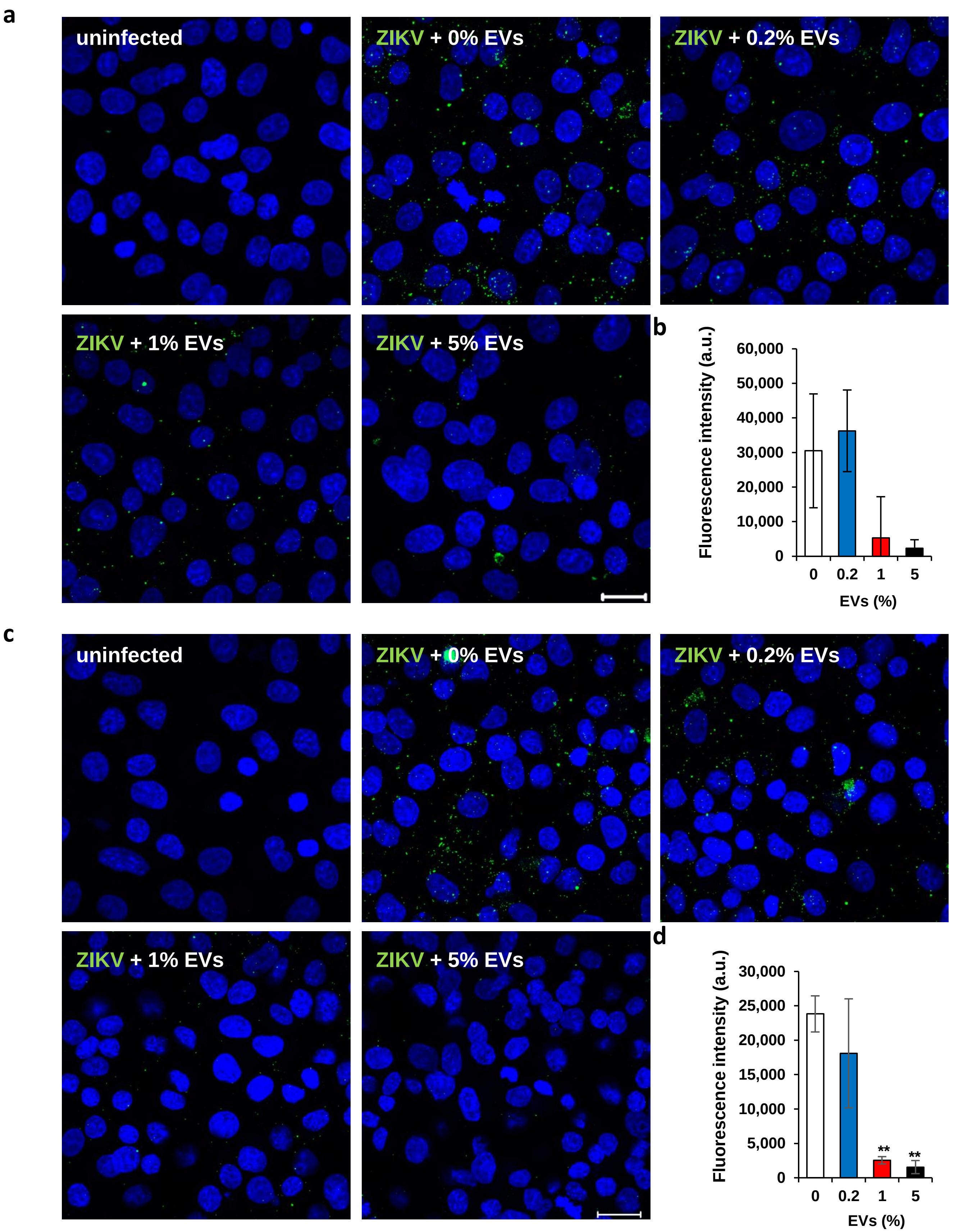

Supplementary Figure 12. Semen derived EVs inhibit ZIKV attachment to cells. a) Vero E6 cells were incubated with ZIKV MR766 in the presence of increasing concentrations of seminal EVs (< 220 nm) for 2 hours at 37°C or at c) 4°C. Cells were then washed, fixed, permeabilized and stained for flavivirus protein E and cell nuclei. A z-stack of 14 confocal microscopy images was taken and combined to a maximum intensity projection. b) Signal intensity of the projections of (a) or d) of (c) was quantified from three z-stacks ± standard deviation (see Fig. 5e, f). a.u.: arbitrary units, ** P<0.001 (by one-way ANOVA with a Bonferroni post-test).

Supplementary Figure 13

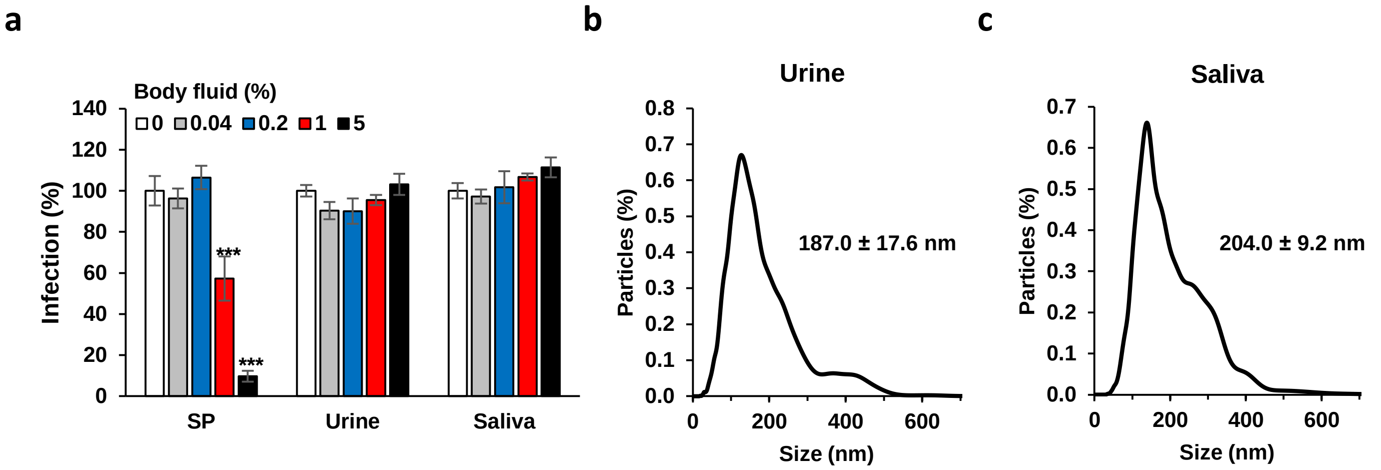

Supplementary Figure 13. EV containing urine and saliva do not affect ZIKV infection. a) 50% SP, urine and saliva was filtered through a 0.2 µm syringe filter. Samples were added to Vero E6 cells at indicated concentrations and incubated for 10 min before cells were inoculated with ZIKV MR766 and incubated for 2 hours followed by a medium change. After 2 days, infection was determined by cell-based ZIKV immunodetection assay that enzymatically quantifies the flavivirus E protein. Average infection rates are normalized to the corresponding infection averages in the absence of the respective body fluid. Data represent average values obtained from triplicate infections ± standard deviations. *** $P<0.0001$ (by one-way ANOVA with a Bonferroni post-test). b) The filtrate of 0.2 µm filtered urine or c) saliva was analyzed for its particle size distribution via nanoparticle tracking analysis using a NanoSight LM10. Data are averages of three tracking measurements for the duration of 60 s.

Supplementary Figure 14

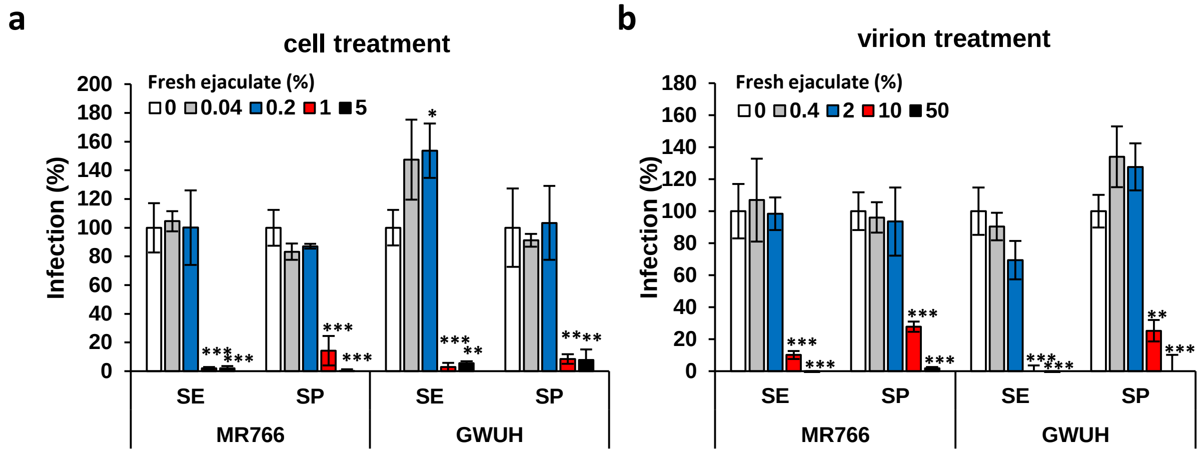

Supplementary Figure 14. A fresh ejaculate inhibits ZIKV infection. a) Fresh ejaculate/semen was allowed to liquefy for ~20 minutes and a fraction of the material was immediately centrifuged (20 min, 20,000 x g) to generate SP. Vero E6 cells were then incubated with the indicated concentrations of SE or SP for 30 min before cells were inoculated with ZIKV. Infection rates were determined two days later by a cell-based ZIKV immunodetection assay. Data are normalized to infection in absence of SE/SP samples and represent average values obtained from triplicate infections ± standard deviations. b) Immediately after liquefaction, ZIKV stocks were incubated with 0, 0.4, 2, 10, 50% of SE or SP for 10 min, and then diluted onto Vero E6 cells. Infection rates were monitored as described in panel (a). ** P<0.001, *** P<0.0001 (by one-way ANOVA with a Bonferroni post-test).

Supplementary Figure 15

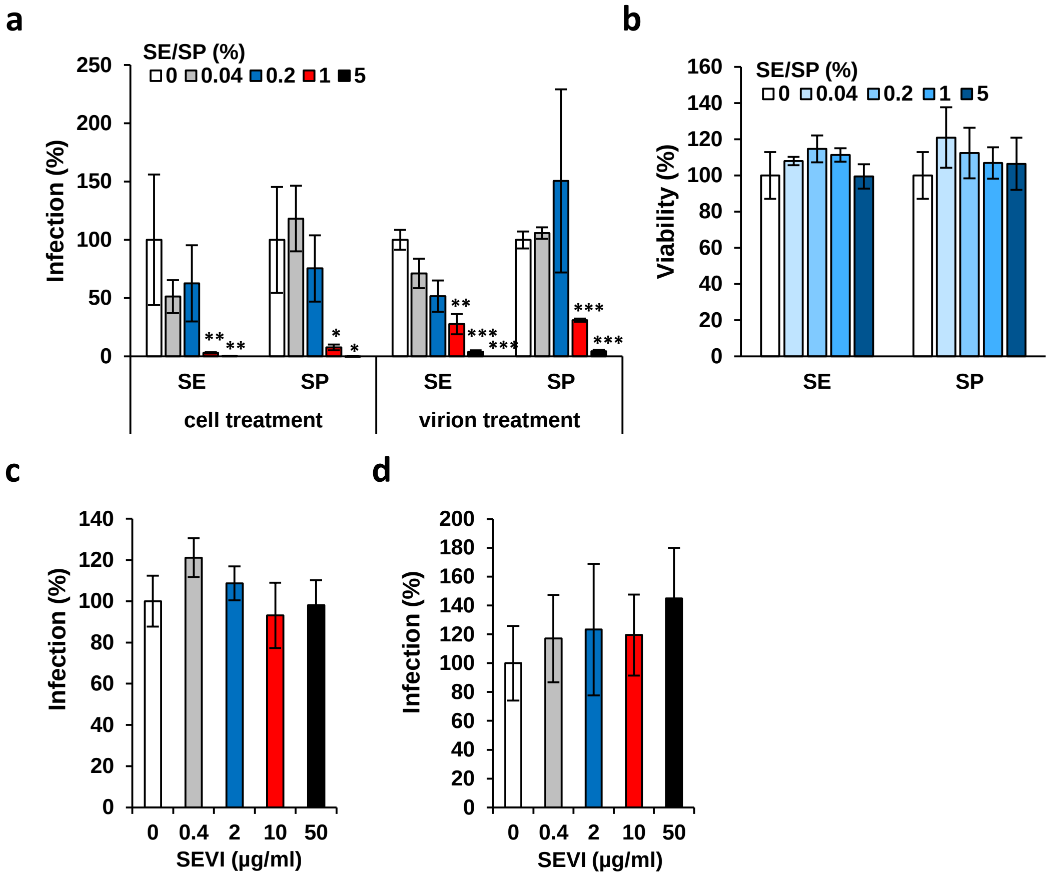

Supplementary Figure 15. The effects of SEVI, SE, and SP on infection by Dengue virus. a) Huh-7 cells were incubated for 10 min with the indicated concentrations of SE or SP ("cell treatment") before inoculation with Renilla luciferase encoding DENV-R2a. Additionally, DENV-R2a was first incubated with 0, 0.4, 2, 10 or 50% of SE or SP for 10 minutes ("virion treatment") and then added to the cells. Indicated SE/SP concentrations correspond to the final concentrations in culture. Two days later infection rates were determined by measuring luciferase activity of cell lysates. b) Huh-7 cells were analyzed for SE and SP cytotoxicity by CellTiter-Glo® luminescent cell viability assay. c) DENV-2 (Thailand/16681/84) or d) DENV-R2a was incubated with increasing concentrations of SEVI and then added to Vero E6 or Huh-7 cells, respectively. Infection was determined two days later by cell-based DENV protein E immunodetection or luciferase assay, respectively. Data are normalized to corresponding averages in the absence of SE, SP, or SEVI and represent average values obtained from triplicates ± standard deviations. * $P<0.01$, ** $P<0.001$, *** $P<0.0001$ (by one-way ANOVA with a Bonferroni post-test).

Supplementary Tables

Supplementary Table 1. ZIKV RT-qPCR primers

Name	Sequence	Provider
**ZIKV RKI-F**	5'-ACGGCYCTYGCTGGAGC-3'	biomers.net, Germany
**ZIKV RKI-R**	5'-GGAATATGACACRCCCTTCAAYCTAAG-3'	biomers.net, Germany
